# The susceptibility of five African *Anopheles* species to *Anabaena* PCC 7120 expressing *Bacillus thuringiensis* subsp. *israelensis* mosquitocidal *cry* genes

**DOI:** 10.1186/1756-3305-5-220

**Published:** 2012-10-04

**Authors:** Irene Ketseoglou, Gustav Bouwer

**Affiliations:** 1 School of Molecular and Cell Biology, University of the Witwatersrand, Private Bag 3, Wits 2050, Johannesburg, South Africa

**Keywords:** Malaria vectors, *Anopheles*, *Bacillus thuringiensis* subsp. *israelensis*, Cry proteins, Cyanobacteria, *Anabaena* sp. PCC 7120, Genetic engineering, Bioassays, Larvicidal activity

## Abstract

**Background:**

Malaria, one of the leading causes of death in Africa, is transmitted by the bite of an infected female *Anopheles* mosquito. Problems associated with the development of resistance to chemical insecticides and concerns about the non-target effects and persistence of chemical insecticides have prompted the development of environmentally friendly mosquito control agents. The aim of this study was to evaluate the larvicidal activity of a genetically engineered cyanobacterium, *Anabaena* PCC 7120#11, against five African *Anopheles* species in laboratory bioassays.

**Findings:**

There were significant differences in the susceptibility of the anopheline species to PCC 7120#11. The ranking of the larvicidal activity of PCC 7120#11 against species in the *An. gambiae* complex was: *An. merus* <*An. arabiensis* <*An. gambiae* <*An. quadriannulatus*, where < indicates a statistically lower LC_50_. The LC_50_ of PCC 7120#11 against the important malaria vectors *An. gambiae* and *An. arabiensis* was 12.3 × 10^5^ cells/ml and 8.10 × 10^5^ cells/ml, respectively. PCC 7120#11 was not effective against *An. funestus*, with less than 50% mortality obtained at concentrations as high as 3.20 × 10^7^ cells/ml.

**Conclusions:**

PCC 7120#11 exhibited good larvicidal activity against larvae of the *An. gambiae* complex, but relatively weak larvicidal activity against *An. funestus*. The study has highlighted the importance of evaluating a novel mosquitocidal agent against a range of malaria vectors so as to obtain a clear understanding of the agent’s spectrum of activity and potential as a vector control agent.

## Background

Species within the genus *Anopheles* (Diptera: Culicidae) play a major role in the transmission of malaria in Africa, in particular mosquitoes from the *An. gambiae* complex and the *An. funestus* group [1,2]. The *An. gambiae* complex contains excellent and efficient vectors of malaria (*An. gambiae s.s.* and *An. arabiensis*), as well as minor vectors (*An. merus*) and non-vectors (*An. quadriannulatus* species A and B) [1]. The *An. funestus* group contains an important vector of malaria, *An. funestus s.s*. [2].

Although chemical insecticides have been used successfully in integrated vector control programs [3], many malaria vector control programs are hampered by the development of resistance of the vectors to chemical insecticides [4-6]. In addition to development of resistance, concerns about the non-target effects and persistence of the chemical insecticides have prompted the development of environmentally friendly control agents and control programs [7].

*Bacillus thuringiensis* subsp. *israelensis* (*Bti*) is a Gram-positive, aerobic, spore-forming, bacterium that produces crystalline inclusions that contain crystal (Cry) or cytolytic (Cyt) proteins that are highly toxic to mosquito larvae [8,9]. Although there is low risk of resistance being developed to *Bti*[10], there are several disadvantages to using *Bti* as a control agent [11,12]. These include its low persistence in the field due to inactivation by UV, ingestion of *Bti* by other aquatic organisms, and the settling of *Bti* from the mosquito larval feeding zone [11-13]. One strategy to overcome some of the disadvantages of *Bti* is to clone the *cry* genes of *Bti* into aquatic microorganisms that: (1) are not toxic to other organisms, (2) inhabit and persist in the larval feeding zone, (3) are used by mosquito larvae as a food source, (4) express Cry proteins at levels that are mosquito larvicidal, and (5) have cell walls that reduce inactivation of the Cry proteins by UV [13-15].

Xiaoqiang *et al*. [15] inserted the *Bti cry4Aa, cry11Aa*, and *p20* genes under the control of two tandem promoters (cyanobacterial constitutive promoter, *P*_*psbA*_, and *Escherichia coli* T7 early promoter, *P*_*A1*_) into a filamentous nitrogen-fixing cyanobacterium, *Anabaena* sp. strain PCC 7120 (PCC 7120). The *Bti* genes are integrated into the chromosome of PCC 7120, resulting in a stable recombinant strain [16]. Laboratory bioassays have shown that the resultant recombinant strain, PCC 7120#11, is a very effective larvicidal agent against *Aedes aegypti*[13,15,16].

To our knowledge, no studies have examined the larvicidal activity of PCC 7120#11 against several African malaria vectors. The aim of this study was, thus, to evaluate the larvicidal activity of PCC 7120#11 against five African *Anopheles* species in order to determine if PCC 7120#11 may have potential as a malaria vector control agent.

## Methods

Larvicidal activity of PCC 7120#11 was determined by laboratory bioassays against four species in the *An. gambiae* complex and one species in the *An. funestus* group. The *An. gambiae* complex species used in the study were (origin, colony name, and colonisation date provided): *An. gambiae s.s.* (Ibadan, Nigeria; NAG; 2001), *An. arabiensis* (Kanyemba, Zimbabwe; KGB; 1975), *An. merus* (KwaZulu-Natal, South Africa; MAF; 1988), and *An. quadriannulatus* species A (Sangwe, Zimbabwe; SANGWE; 1998). The species from the *An. funestus* group that was used in the study was *An. funestus s.s.* (Maputo, Mozambique; FUMOZ; 2000).

The anopheline mosquito species were obtained from colonies maintained at the National Institute of Communicable Diseases (Johannesburg, South Africa). Since the activity of PCC 7120#11 against *A. aegypti* had been previously evaluated [15], we included it as a control in the bioassays. The *A. aegypti* larvae were obtained from the South African Bureau of Standards (Pretoria, South Africa).

PCC 7120 and PCC 7120#11, were cultured in BG-11 medium [15], at 30°C under continuous illumination (2000 lux) with constant agitation [13,16]. The PCC 7120 and PCC 7120#11 cells were harvested by differential centrifugation and the cell concentration was determined by haemocytometer counts. Two millilitres of the appropriate dilution (covering an in-cup concentration range of 1.00 × 10^4^ to 3.20 × 10^7^ cells/ml) of either PCC 7120 or PCC 7120#11 was added to 130 ml plastic cups that contained 98 ml sterile distilled water and 20 third-instar mosquito larvae. In the case of *An. merus*, a sterile 5 M saline solution was used instead of sterile distilled water.

Larvicidal activity was determined 24 hours post-inoculation, with larvae presumed dead if they did not move when prodded. An untreated control (sterile distilled water) was included in the bioassays. If mortality in the controls (PCC 7120 and untreated) exceeded 5%, the test was discarded and repeated. Each bioassay was repeated in triplicate on different days. The lethal concentration (LC_50_ and LC_90_) for each species was determined by probit analysis [17]. For each species, probit analysis was based on the mortality data obtained from five PCC 7120#11 concentrations.

## Results and discussion

The concentration-mortality data for the mosquito species are summarised in Table 1. The heterogeneity factors for the different mosquito species evaluated were all less than one, indicating a good fit of the concentration-mortality data to the probit model [18].

**Table 1:** **Probit analysis of concentration-mortality data for *Anabaena *PCC 7120#11 against third instar mosquito larvae**

**Species**	**LC**_**50**_**(10**^**5**^cells/ml)^*^	**LC**_**90**_**(10**^**5**^cells/ml)^*^	**Slope ± SE**^**†**^	**Heterogeneity**^**‡**^
*A. aegypti*	1.42 (1.12-1.76)^a^	8.21 (6.05-12.3)^a^	1.70±0.15^a^	0.75
*An. merus*	3.90 (3.58-4.17)^b^	9.30 (8.0-11.4)^a^	3.37±0.31^b^	0.62
*An. arabiensis*	8.10 (7.62-8.56)^c^	14.3 (12.8-16.5)^b^	5.18±0.46^c^	0.19
*An. gambiae*	12.3 (11.4-13.3)^d^	35.1 (29.5-44.9)^c^	2.81±0.24^d^	0.49
*An. quadriannulatus*	15.7 (14.3-17.3)^e^	43.0 (35.9-54.9)^c^	2.93±0.26^bd^	0.92
*An. funestus*	N.D.^§^	N.D.	N.D.	N.D.

* Values in brackets show the 95% fiducial limits (FLs). Values in a column followed by the same letters are not significantly different (overlapping 95% FLs).

^†^ Slope ± standard error. Values followed by the same letters are not significantly different (*p* > 0.05).

^‡^ Heterogeneity factor = χ^2^ / d.f. (degrees of freedom).

^§^ Not determined. Less than 50% mortality was obtained even at concentrations as high as 3.2 × 10^7^ cells/ml.

The concentration-mortality regression slopes indicate the variability in response to a toxin within the vector population being examined [19]. In this study, *An. arabiensis* had a significantly steeper slope than the other species evaluated, suggesting that *An. arabiensis* had lower response variability or reduced heterogeneity in its population compared to the other mosquito species examined. The shallower slopes of the concentration-mortality regression lines obtained for *An. gambiae* and *An. quadriannulatus* mean that there are larger differences between the LC_50_ and LC_90_ values for these species than for the other anopheline species evaluated.

Although slight variation in LC_50_s between studies may be expected due to differences in experimental conditions such as rearing conditions of the larvae and natural variations in the larval populations [20], the susceptibility of *A. aegypti* larvae to PCC 7120#11 in this study was comparable to that (LC_50_ of 0.9 × 10^5^ cells/ml) previously reported by Xiaoqiang *et al*. [15]. However, the LC_50_ value of PCC 7120#11 against *A. aegypti* larvae was significantly lower than those of the anopheline species evaluated (Table 1). The decreased susceptibility of anopheline larvae compared to *A. aegypti* larvae may be due to differences in feeding behaviour [21]; *Anopheles* larvae (surface feeders) may ingest fewer PCC 7120#11 cells than *A. aegypti* larvae that feed at all levels in the water column.

The LC_50_ values differed significantly between the anopheline species examined, with PCC 7120#11 being most effective against *An. merus* larvae (Table 1). On the basis of LC_50_ ratios, the larvae of *An. quadriannulatus* were 4-fold less susceptible to PCC 7120#11 than the larvae of *An. merus*. PCC 7120#11 had relatively weak larvicidal activity against *An. funestus*, even at concentrations as high 3.2 × 10^7^ cells/ml*.* The LC_90_ values for *An. merus* and *A. aegypti* were significantly lower than the LC_90_ values for *An. arabiensis*, *An. quadriannulatus*, or *An. gambiae* (Table 1). Furthermore, the LC_90_ for *An. arabiensis* was significantly lower than that of *An. quadriannulatus* or *An. gambiae* (Table 1).

The significant differences in susceptibility to PCC 7120#11 between the anopheline species may reflect species-specific differences in one or more of the steps in the ingestion-to-toxicity process. For example, differences in the ingestion rate or efficiency of digestion of PCC 7120#11 cell walls by the different anopheline species may significantly affect the concentration of Cry proteins in the larval midgut. However, preliminary evaluations (unpublished data) did not show marked differences in the ingestion or digestion rates between *An. funestus* and *An. arabiensis*.

A factor to consider in interpreting the significant differences in susceptibility between *An. funestus* and species of the *An. gambiae* complex is the combination of the toxins present in PCC 7120#11. *Bti*, which naturally produces crystals that contain the toxins Cry4Aa, Cry4Ba, Cry10Aa, Cry11Aa, Cyt1Aa and Cyt2Ba [9,22], has higher larvicidal activity against mosquitoes than recombinant clones containing a subset of toxins [9,23,24]. In a previous study [25], *Bti* strain HD522 was evaluated against the same anopheline species, with *An. funestus* larvae having an LC_50_ that was statistically similar to the LC_50_ values obtained for *An. arabiensis*, *An. gambiae*, and *An. merus* larvae. The comparatively low larvicidal activity of PCC 7120#11 against *An. funestus* could be due to the absence of the other *Bti* Cry and Cyt proteins in PCC 7120#11, which produces only the Cry4Aa and Cry11Aa proteins. Since the larvicidal activity of Cry proteins is often correlated with the high affinity binding of the proteins to specific membrane-bound receptors in the larval midgut [26,27], the susceptibility differences could be due to inherent differences between the species in the structure or density of midgut receptors for Cry4Aa or Cry11Aa. In this context, it is noteworthy that Cyt1A is known to synergize Cry11A toxicity by functioning as a membrane-bound receptor [28]. Further research is required to determine which combinations of the Cry and Cyt proteins would result in the highest larvicidal activity against *An. funestus* larvae.

PCC 7120#11 displayed good larvicidal activity against key malaria vectors, including *An. gambiae* and *An. arabiensis*. Although PCC 7120#11 displayed comparatively weak larvicidal activity against *An. funestus* at economically practical concentrations, PCC 7120#11 may have potential as a larvicidal agent in geographic regions where *An. funestus* is not the predominant vector. Alternatively, PCC 7120#11 could be applied as a larvicidal agent as part of an integrated vector control program that targets *An. funestus* adults and the larvae of other malaria vectors. However, before PCC 7120#11 can used in vector control programs, its effects on non-target organisms and its persistence in aquatic environments would have to be comprehensively evaluated.

## Competing interests

The authors declare that they have no competing interests.

## Authors’ contributions

Conceived the idea: GB. Performed the experiment and analyzed the data: IK and GB. Contributed reagents/materials/analysis tools: GB. Wrote the manuscript: IK. Clarified the manuscript: GB. All authors read and approved the final manuscript.
